# Capturing indirect genetic effects on phenotypic variability: Competition meets canalization

**DOI:** 10.1111/eva.13353

**Published:** 2022-03-08

**Authors:** Jovana Marjanovic, Han A. Mulder, Lars Rönnegård, Dirk‐Jan de Koning, Piter Bijma

**Affiliations:** ^1^ Animal Breeding and Genomics Wageningen University and Research Wageningen The Netherlands; ^2^ 3317 Department of Animal Breeding and Genetics Swedish University of Agricultural Sciences Uppsala Sweden; ^3^ 3317 Department of Information Technology Dalarna University Falun Sweden

**Keywords:** canalization, competition, IGE, indirect genetic effects, inherited variability, statistical models

## Abstract

Phenotypic variability of a genotype is relevant both in natural and domestic populations. In the past two decades, variability has been studied as a heritable quantitative genetic trait in its own right, often referred to as inherited variability or environmental canalization. So far, studies on inherited variability have only considered genetic effects of the focal individual, that is, direct genetic effects on inherited variability. Observations from aquaculture populations and some plants, however, suggest that an additional source of genetic variation in inherited variability may be generated through competition. Social interactions, such as competition, are often a source of Indirect Genetic Effects (IGE). An IGE is a heritable effect of an individual on the trait value of another individual. IGEs may substantially affect heritable variation underlying the trait, and the direction and magnitude of response to selection. To understand the contribution of IGEs to evolution of environmental canalization in natural populations, and to exploit such inherited variability in animal and plant breeding, we need statistical models to capture this effect. To our knowledge, it is unknown to what extent the current statistical models commonly used for IGE and inherited variability capture the effect of competition on inherited variability. Here, we investigate the potential of current statistical models for inherited variability and trait values, to capture the direct and indirect genetic effects of competition on variability. Our results show that a direct model of inherited variability almost entirely captures the genetic sensitivity of individuals to competition, whereas an indirect model of inherited variability captures the cooperative genetic effects of individuals on their partners. Models for trait levels, however, capture only a small part of the genetic effects of competition. The estimation of direct and indirect genetic effects of competition, therefore, is possible with models for inherited variability but may require a two‐step analysis.

## 1 INTRODUCTION

Some genotypes show less variable phenotypes compared to others in response to perturbations in the genome or environment. The genetic mechanism that leads to insensitivity of a phenotype to genetic and environmental perturbations is known as “canalization” (Waddington, 1942). Evolution of canalization is often associated with stabilizing natural selection for an optimal phenotype, as such selection favors mechanisms that reduce variance around the optimum so that more extreme phenotypes do not occur (Edgell et al., 2009; Flatt, 2005; Waddington, 1942; Wagner et al., 1997). Long‐term stabilizing selection of a trait is therefore expected to reduce phenotypic variation.

Depending on the source of perturbation, canalization can be either genetic or environmental. In the following, we focus on environmental canalization. Environmental canalization is commonly inferred from the size of the environmental variance ($V_{e}$) of a genotype. In other words, genotypes that produce more stable phenotypes have lower $V_{e}$, and a decrease of $V_{e}$ due to selection indicates canalization (Flatt, 2005; Gibson & Bradley, 1974; Wagner et al., 1997).

Phenotypic variability of a genotype is relevant not only in natural populations but also in agriculture. In animal and crop production, low variability of traits is often of economic importance. In the pig industry, for example, excessive variability in size and weight of animals is penalized by slaughterhouses, so that delivering animals within a preferred range has an economic benefit (Hennessy, 2005; Mulder et al., 2008). In aquaculture, fish that deviate too much from the average size are usually not sold, which reduces revenues (Khaw et al., 2016; Marjanovic et al., 2016). Low $V_{e}$ in crops is desirable, as it indicates stability against unpredictable conditions (Edwards & Jannink, 2006). Selection for trait uniformity in animal and plant breeding is an analogy of evolution of canalization in natural populations.

The phenotypic variability of a genotype, measured either repeatedly on the same individual or on multiple individuals belonging to the same family, has been studied as a quantitative trait in its own right. This concept was first introduced by Waddington (1942) and has been an integrative part of quantitative genetics ever since, with growing interest over the last two decades, largely due to the development of methods to estimate the genetic variance in $V_{e}$ (Mulder et al., 2009; Rönnegård et al., 2010; SanCristobal‐Gaudy et al., 1998; Sorensen & Waagepetersen, 1942, 2003). Inheritance of the phenotypic variability of a genotype is often referred to as “inherited variability” or “heritable variation in environmental variance” (Hill & Mulder, 2010; Mulder et al., 2007; SanCristobal‐Gaudy et al., 1998). There is strong evidence of genetic variation in $V_{e}$. The study by Mackay and Lyman (2005), who compared bristle number of different isofemale lines of Drosophila, is one of the best evidences that genotypes differ in $V_{e}$, that is, that environmental canalization has a genetic component. In addition, a number of studies in plant and animal populations showed that variability often has a substantial genetic component (reviewed by Hill & Mulder, 2010 and Iung et al., 2020).

The majority of studies focusing on the inheritance of quantitative traits consider only the direct genetic effects of an individuals’ own genes (DGE) on the trait value of the individual itself. However, most individuals are not solitary but rather social organisms, and with social interactions, such as competition and cooperation, the trait value of an individual may be influenced not only by the individuals’ own genes but also by the genes of its social partner. This heritable effect of a social partner on the trait values of the focal individual is known as an indirect genetic effect (IGE; referred to as associative effects in Griffing, 1967). IGEs have been studied in animals (e.g., Ellen et al., 2014), plants (e.g., Brotherstone et al., 2011; Mutic & Wolf, 2007), and microorganisms (Crespi, 2001), and both in natural (e.g., Wilson et al., 2011) and in domestic populations (e.g., Khaw et al., 2016; Muir, 1996). A number of studies have shown that social interactions can contribute substantially to heritable variation underlying a trait, and may change both the magnitude and the direction of response to selection (Bijma, 2011; Bijma et al., 2007; Ellen et al., 2007; Griffing, 1976, 1977; Hamilton, 1964a, 1964b).

So far, social interactions have been studied mainly in relation to their effects on trait values of individuals. However, in aquaculture populations, it has been observed that competition for feed and formation of a social hierarchy also increases the variation of trait values among individuals (Cutts et al., 1998; Hart & Salvanes, 2000; Jobling, 1995). Phenotypic studies show that populations displaying less competition tend to grow more uniformly and have higher average performance (Cutts et al., 1998; Hart & Salvanes, 2000; Jobling, 1995). These observations suggest that phenotypic variability may also be affected by social interactions, with IGEs harboring genetic variation in variability that has been overlooked so far. Previously, studies in Atlantic salmon (Sonesson et al., 2013), rainbow trout (Janhunen et al., 2012; Sae‐Lim et al., 2015), and Nile tilapia (Khaw et al., 2016; Marjanovic et al., 2016) identified a large direct genetic component in the variability of body weight.

The relationship between competition and phenotypic variability is not unique for aquaculture but can also be observed in plants. Plant breeders have successfully improved the productivity of crops by selecting, partly unintentionally, less competitive phenotypes, which has resulted in more uniform crops (Austin et al., 1980; Denison et al., 2003; Donald, 1968).

Until recently, we lacked the tools to investigate whether IGEs also contribute to genetic variation in variability. IGE‐models come in two types; variance‐component models and trait‐based models (Griffing, 1967; Moore et al., 1997; reviewed by Bijma, 2014 and McGlothlin & Brodie, 2009). Variance component models cannot explain the observed relationship between competition and variability because phenotypic variance is independent of the average level of the IGE. Trait‐based models, in contrast, lead to a relationship between competition and variability, but on the population level, this relationship is identical for competition and cooperation, which does not reflect the pattern observed in real populations. On the other hand, current models of inherited variability treat variability as a property of a single individual, ignoring the component due to competition.

We recently proposed a quantitative genetic model that allows for a relationship between IGEs and inherited variability (Marjanovic et al., 2018). In this model, competition between social partners leads to divergence of their phenotypes (e.g., body weight) over their lifetime. Hence, the model allows for indirect genetic effects to lead to differences in variability of trait values, on both group and population level, similar to observations in real populations.

To understand the contribution of IGEs to the evolution of environmental canalization in natural populations, and to exploit such inherited variability in animal and plant breeding, we need statistical models to capture this phenomenon. The model of Marjanovic et al. (2018) can be used to estimate effects of competition, but it requires time‐series data, which are often not available. The use of existing statistical models for IGE and inherited variability applied to a single phenotype, such as the final phenotype of a time series, would allow the study of a much broader range of cases. However, to our knowledge, it is unknown to what extent such models capture the effect of competition on inherited variability.

Here, we investigate the potential of existing statistical models for inherited variability and for trait values, to capture the direct and indirect genetic effects of competition on variability. To address this issue, we conducted a simulation study in which competition between social partners (i.e., IGEs) leads to inherited variability of trait values, using the model of Marjanovic et al. (2018). Subsequently, we analyzed these data with four models. The ability of those models to capture direct and indirect genetic effects on variability was tested by comparing estimated genetic effects from each of the models with simulated direct breeding values for trait level, and with direct and indirect breeding values for competition.

## 2 MATERIALS AND METHODS

### 2.1 Quantitative genetic model

In this section, we summarize the quantitative genetic model of Marjanovic et al. (2018) that integrates IGEs and inherited variability. This model was used to simulate a population in which competition affects variability. We use individual growth rate of fish, modeled over time, as an example to illustrate the model.

In this model, we consider groups of two individuals. Each individual is both a focal individual in the model for its own phenotype, and a social partner in the model for the phenotype of its group mate. In aquaculture, the growth of individuals is affected by the difference in body weight between interacting individuals, with higher body weight giving a competitive advantage to an individual in terms of growth (Doyle & Talbot, 1986). Therefore, the phenotypic value for the growth rate of the focal individual is affected by the ordinary direct genetic and environmental effects of the focal individual itself, and by the difference in body weight between the focal individual and its social partner. The degree to which the difference in body weight affects the phenotype of an individual is measured by a regression coefficient *b*,

(1) $$P_{t,i}‐P_{t‐1,\;i}\;=\mu_{\mathrm{GR}}+A_{\mathrm{GR},i}+E_{p,\;\mathrm{GR},i}+E_{t,\mathrm{GR},i}+b_{\mathrm{ij}}\left(P_{t‐1,j}‐P_{t‐1,i}\right)$$

where $P_{t,\;i}$ is the body weight of focal individual *i* at time point *t*, $P_{t‐1,\;i}$ is the body weight of *i* at the previous time point, $\mu_{\mathrm{GR}}$ is the mean growth rate of the population, $A_{\mathrm{GR},i}$ is a (direct) breeding value for the growth rate of individual *i*, $E_{p,\;\mathrm{GR},i}$ and $E_{t,\mathrm{GR},i}$ are permanent and temporary non‐heritable (“environmental”) effects of individual *i*, and $b_{\mathrm{ij}}$ is a regression coefficient.

The absolute value of $b_{\mathrm{ij}}$ describes the strength of the social interaction. The sign of *b* is a measure of cooperation, where a negative *b* indicates competition, while a positive *b* indicates cooperation. A negative *b*, for example, indicates that an individual grows slower when its partner has a higher body $\left(P_{t‐1,j}‐P_{t‐1,i}>0\right)$.

In this model, *b* is not a fixed parameter, but a composite genetic trait that can evolve over generations. The *b* exhibits genetic variation due to a direct genetic effect of the focal individual ($A_{D,i}$), representing “genetic resistance to competition,” and an indirect genetic effect of its social partner, representing the “genetic cooperative effect” ($A_{I,j}$). Hence, the model allows for variation among individuals in sensitivity to competition, so that some individuals may suffer less from the presence of a large social partner than others. Similarly, the model allows for variation among individuals in competitive effect. Some individuals may be large at the expense of their group mate, whereas other large individuals may not suppress the growth of their social partner. Thus, for focal individual *i* with social partner *j*, the regression coefficient $b_{\mathrm{ij}}$ is given by

(2) $$b_{\mathrm{ij}}=\bar{b}+A_{D,i}+E_{D,i}+A_{I,j}+E_{I,j}\;$$

where $\bar{b}$ represents the average regression coefficient, which is a population parameter that is negative under competition and positive under cooperation. The $A_{D,i}$ and $E_{D,i}$ are the direct genetic and environmental effect of individual *i* on $b_{\mathrm{ij}}$, while $A_{I,j}$ and $E_{I,j}$ are the indirect genetic and environmental effect of individual *j* on $b_{\mathrm{ij}}$. As common in quantitative genetics, *A* and *E* are defined relative to a mean of zero. A negative value of $A_{D}$ indicates that the individual is sensitive to competition (as compared to an average individual), while an individual with positive $A_{D}$ is resistant to competition. Similarly, an individual with negative $A_{I}$ is competitive, in the sense that it suppresses the body weight of its partner by a relatively large amount, while an individual with positive $A_{I}$ is cooperative. Note that *b* is non‐symmetric, that is, $b_{\mathrm{ij}}\neq b_{\mathrm{ji}}$, as individuals may differ in their breeding values for *b*. In other words, an individual that is strongly affected by its social partner, does not necessarily also have a strong effect on its social partner.

Therefore, in the total model (Equations 1 and 2), there are three breeding values – an ordinary direct breeding value for growth rate $\left(A_{\mathrm{GR}}\right)$, a direct breeding value for *b* ($A_{D}$) representing genetic resistance to competition, and an indirect breeding value for $b\left(A_{I}\right)$ representing a genetic cooperative effect.

### 2.2 Simulation

#### 2.2.1 Population structure

We simulated a family‐structured population using the model presented above (Equations 1 and 2). Our objective was to test whether currently available models for IGE and inherited variability capture the effect of IGE on variability, rather than to investigate the statistical power of those models. For this reason, we simulated large data sets to avoid that limited power would blur the results.

We first simulated a base population of 100 sires and 10,000 dams, all unrelated. Each animal in the base population was assigned a breeding value for growth rate, and a direct and indirect breeding value for *b*, drawn from a multivariate normal distribution. Next, the offspring population was created by mating each sire with 100 randomly chosen dams. Body weight records were simulated on the offspring generation only. For the simulation of data on trait levels, each dam produced 10 offspring, resulting in 1000 offspring per sire, and a total of 100,000 offspring. For the simulation of data on inherited variability, we simulated a larger data set because analysis of variability was performed on records grouped by family (see below). Thus, to create records on the variability of body weight, each dam produced 100 offspring, resulting in 10,000 offspring per sire, and a total of 1 million offspring.

The breeding values for growth rate and direct and indirect breeding values for *b* of the offspring were simulated as the average breeding value of the sire and dam, plus a Mendelian sampling term drawn from $N\left(\left[\begin{array}{c} 0\\ 0\\ 0 \end{array}\right]\right.,\;\frac{1}{2}\left.\left[\begin{array}{ccc} \sigma_{A_{\mathrm{GR}}}^{2} & 0 & 0\\ 0 & \sigma_{A_{D}}^{2} & 0\\ 0 & 0 & \sigma_{A_{I}}^{2} \end{array}\right]\right)$. In addition, each offspring was assigned a permanent and temporary environmental effect on body weight, and a direct and indirect environmental effect on *b*. These were drawn from $N\;\left.\left(\left[\begin{array}{c} 0\\ 0\\ 0\\ 0 \end{array}\right]\right.,\left[\begin{array}{cccc} \sigma_{E_{p,\mathrm{GR}}}^{2} & 0 & 0 & 0\\ 0 & \sigma_{E_{t,\mathrm{GR}}}^{2} & 0 & 0\\ 0 & 0 & \sigma_{E_{D}}^{2} & 0\\ 0 & 0 & 0 & \sigma_{E_{I}}^{2} \end{array}\right]\right)$. In the default scenario, the genetic and environmental covariances were all set to 0.

Groups of two members were created by randomly assigning a social partner to each offspring, which resulted in 50,000 groups for the analysis of trait levels, and 500,000 groups for the analysis of trait variability. Subsequently, phenotypes for 10‐time points were obtained for all individuals by using Equations 1 and 2. Body weight at the last time point was used as the trait of interest and may, for example, reflect harvest weight in fish. Simulations were conducted using the R software (R Development Core Team, 2011).

#### 2.2.2 Parameters

The parameters were chosen to represent the growth of fish as an example. Table 1 shows the parameters used in the simulations. Starting weight of the individuals was set to 10 g. Mean growth rate ($\mu_{\mathrm{GR}}$) was also 10 g. The genetic standard deviation of growth rate ($\sigma_{A_{\mathrm{GR}}}$) was set to 1 g. (See Marjanovic et al. (2018) for examples of the typical behavior of populations for these parameter values). Repeatability was set to 0.7, and heritability of growth rate to 0.5 in the absence of social interactions (*b* = 0). Phenotypic variance was calculated as $\sigma_{P}^{2}$ = $\sigma_{A_{\mathrm{GR}}}^{2}/h^{2}$ and was equal to 2 g^2^, permanent environmental effect on growth ($\sigma_{E_{p,\mathrm{GR}}}^{2}$) as 0.2 $\sigma_{P}^{2}$ = 0.4 g^2^ and temporary environmental effect ($\sigma_{E_{t,\mathrm{GR}}}^{2}$) as 0.3 $\sigma_{P}^{2}$ = 0.6 g^2^ (Table 1).

**TABLE 1:** Parameters used in the simulation

Parameters	Default values	Alternate values
Mean growth rate, $\mu_{\mathrm{GR}}$	10 g	
Starting weight	10 g	
Genetic standard deviation for growth rate, $\sigma_{A_{\mathrm{GR}}}$	1 g	3 g or 0.3 g
Cooperation effect, $\bar{b}$	−0.05, 0, or 0.05	
Direct and indirect genetic standard deviation, $\sigma_{A_{D}}=\sigma_{A_{I}}$	0.015	0.045 or 0.005
Direct and indirect environmental standard deviation, $\sigma_{E_{D}}=\sigma_{E_{I}}$	0.015	
Phenotypic variance, $\sigma_{P_{\mathrm{GR}}}^{2}$ ^a^	2 g	18 g or 0.18 g
Permanent environmental variance, $\sigma_{E_{p,\mathrm{GR}}}^{2}$ ^b^	0.4 g	3.6 g or 0.036 g
Temporary environmental variance, $\sigma_{E_{t,\mathrm{GR}}}^{2}$ ^b^	0.6 g	5.4 g or 0.054 g

^a^ $\sigma_{P_{\mathrm{GR}}}^{2}$ was calculated assuming *b* = 0 i.e., as $\sigma_{P_{\mathrm{GR}}}^{2}=\frac{\sigma_{A_{\mathrm{GR}}}^{2}}{h}$, where h=0.5.

^b^ $\sigma_{E_{p,\mathrm{GR}}}^{2}$ was calculated as 0.2 $\sigma_{P_{\mathrm{GR}}}^{2}$, and $\sigma_{E_{t,\mathrm{GR}}}^{2}$ as 0.3 $\sigma_{P_{\mathrm{GR}}}^{2}$.

The $\bar{b}$ values used in the simulation were −0.05 (competition), 0 (no social interaction), or 0.05 (cooperation). The total standard deviation of $\bar{b}$ was set to 60% of 0.05; $\sigma_{b}=0.03$. Therefore, standard deviations of genetic and environmental components of *b* had to satisfy $\sqrt{\sigma_{A_{D}}^{2}+\sigma_{A_{I}}^{2}+\sigma_{E_{D}}^{2}+\sigma_{E_{I}}^{2}}=0.03$. All standard deviations were assumed equal; hence, each of them had a value of 0.015 (Table 1).

In addition to the default values of $\sigma_{A_{D}},\;\sigma_{A_{I}},$ and $\sigma_{A_{\mathrm{GR}}}$, we also simulated data where these values were 3× larger or 3× smaller (Table 1). These values were used to test the effect of the magnitude of the genetic variance on the estimates. In total, we tested 21 scenarios with different values of $\sigma_{A_{D}},\;\sigma_{A_{I}}$, $\sigma_{A_{\mathrm{GR}}},$ and *b* (Table 2).

**TABLE 2:** Scenarios

	Scenario^a^	$\bar{b}$ effect^b^	$\sigma_{A_{D}}$	$\sigma_{A_{I}}$	$\sigma_{A_{\mathrm{GR}}}$
Default scenario	1	Competition	0.015	0.015	1
	2	Neutral	0.015	0.015	1
	3	Cooperation	0.015	0.015	1
Different $\sigma_{A_{D}}$	4	Competition	**0.045**	0.015	1
	5	Neutral	**0.045**	0.015	1
	6	Cooperation	**0.045**	0.015	1
	7	Competition	**0.005**	0.015	1
	8	Neutral	**0.005**	0.015	1
	9	Cooperation	**0.005**	0.015	1
Different $\sigma_{A_{I}}$	10	Competition	0.015	**0.045**	1
	11	Neutral	0.015	**0.045**	1
	12	Cooperation	0.015	**0.045**	1
	13	Competition	0.015	**0.005**	1
	14	Neutral	0.015	**0.005**	1
	15	Cooperation	0.015	**0.005**	1
Different $\sigma_{A_{\mathrm{GR}}}$	16	Competition	0.015	0.015	**3**
	17	Neutral	0.015	0.015	**3**
	18	Cooperation	0.015	0.015	**3**
	19	Competition	0.015	0.015	**0.3**
	20	Neutral	0.015	0.015	**0.3**
	21	Cooperation	0.015	0.015	**0.3**

^a^ Parameter values that differ from those in default scenario are given in bold.

^b^ Competition corresponds to $\bar{b}$ of −0.05; Neutral corresponds to $\bar{b}$ of 0; Cooperation corresponds to $\bar{b}$ of +0.05.

Finally, we investigated how a non‐zero genetic correlation affects estimated correlations, by simulating data with correlations of −0.5 or +0.5 between $\sigma_{A_{D}},\;\sigma_{A_{I}},$ $\sigma_{A_{\mathrm{GR}}}$, and default values for the other parameters.

For the analysis of inherited variability, each scenario had 100 replicates. For the analysis of levels of a trait, each scenario had 10 replicates.

### 2.3 Statistical models

We estimated genetic effects for body weight at the last time point and its variability, using two models for each trait. For inherited variability, these were (i) a direct sire model, and (ii) an indirect sire model. For the trait, these were (iii) a direct sire–dam model, and (iv) an indirect sire–dam model. We used sire models and data grouped by family for inherited variability, because this is a simple and robust approach to estimate genetic parameters and genetic effects for $V_{E}$, and avoids the need for complex models such as double hierarchical generalized linear models (Rönnegård et al., 2010). For all four models, genetic effects were estimated using residual maximum likelihood (REML) implemented in ASReml 4.1 software (Gilmour et al., 2015). Subsequently, we estimated Pearson correlations between the estimated genetic effects from each model and each of the simulated breeding values. Estimated genetic effects from sire models were correlated with simulated breeding values of sires, while estimated genetic effects from sire–dam models were correlated with simulated breeding values of both sires and dams. Table 3 gives an overview of the estimated correlations. Models are explained in detail below.

**TABLE 3:**
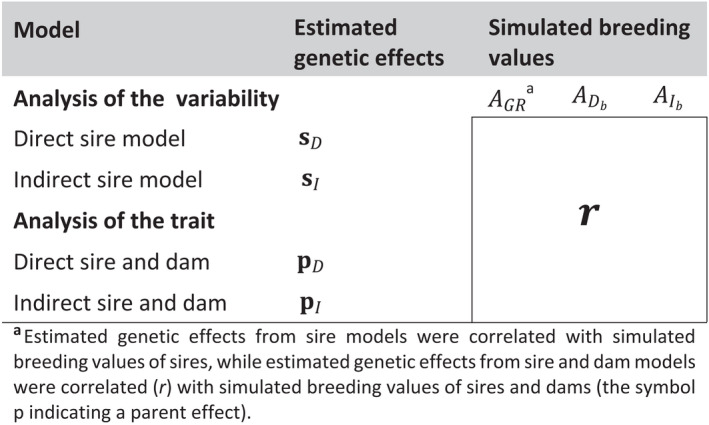
Overview of estimated correlations between estimated and simulated breeding values

#### 2.3.1 Direct sire model for inherited variability

As a measure of the direct component of inherited variability, we used the log‐transformed within‐family variance of body weight. Log transformed within‐family variance of one full‐sib family was treated as a trait of the sire, so that each sire had 100 observations of within‐family variance, each based on 100 offspring per sire–dam combination. This model corresponds to an ordinary model for inherited variability (Rowe et al., 2006), and gives estimates of half of the direct breeding values of a sire for inherited variability ($\left(s_{D}\right)$. The model was:

$$y_{v,D}=\mu +Z_{D_{s}}s_{D}+e,$$

where $y_{v,D}$ is the vector of log‐transformed within‐family variance of body weight, *µ* is the overall mean, $s_{D}$ is a vector of direct random genetic effects of sires, with $s_{D}$ ~ N(**0**, $\sigma_{S_{D}}^{2}$), where $\sigma_{S_{D}}^{2}$ is the direct sire variance, $Z_{D_{s}}$ is an incidence matrix linking observations to sires, and e is the vector of random residuals, with e ~ N(**0**, $\sigma_{e}^{2}$).

#### 2.3.2 Indirect sire model for inherited variability

Indirect genetic effects are expressed in the phenotypes of social partners. Therefore, to estimate indirect random genetic effects of sires for variability $\left(s_{I}\right)$, we used the log‐transformed variance of body weight of the group mates of full‐sib families descending from the sire. Thus, each sire had 100 records, which were the log‐transformed variance of body weight of the group mates of each of the 100 families produced by a sire. The model was:

$$y_{v,I}=\mu +Z_{I_{s}}s_{I}+e,$$

where $y_{v,I}$ is the vector of log‐transformed variance of body weight of the group mates of the full‐sib families descending from the sire, *µ* is the overall mean, $s_{I}$ is the vector of indirect random genetic effects of a sire, with ($s_{I})$ ~ N(**0**, $\sigma_{s_{I}}^{2}$), where $\sigma_{s_{I}}^{2}$ is the indirect sire variance for variability, $Z_{I_{s}}$ is an incidence matrix linking observations to sires, and e is the vector of random residuals, with (e) ~ N(**0**, $\sigma_{e}^{2}$).

#### 2.3.3 Direct sire–dam model for the trait

Here, we use an ordinary sire–dam model, which assumes equal genetic variance for sires and dams. The model was as follows:

$$y_{t,D}=\mu +Z_{D_{p}}p_{D}+e,$$

where $y_{t,D}$ is the vector of individual body weight records of offspring, *µ* is the overall mean, $p_{D}$ is the vector of direct random genetic effects of sires and dams (“parents”), with $p_{D}$ ~ N(**0**, $\sigma_{p_{D}}^{2}$), where $\sigma_{p_{D}}^{2}$ is the direct sire–dam variance, $Z_{D_{p}}$ is an incidence matrix linking observations to parents, and has a “1” in the column for the sire and in the column for the dam of the offspring producing the record, and e is the vector of random residuals, with e ~ N(**0**, $\sigma_{e}^{2}$). This model gives estimates of half of the direct breeding values of a parent for body weight $\left(p_{D}\right)$.

#### 2.3.4 Indirect sire–dam model for the trait

In this model, we link the observation on an individual to the sire and dam of its group mate. The model was:

$$y_{t,I}=\mu +Z_{I_{p}}p_{I}+e,$$

where $y_{t,I}$ is the vector of individual body weight records of individuals, *µ* is the overall mean, $p_{I}$ is the vector of indirect random genetic effects of the parents of the group mate of the focal individual, with $p_{I}$ ~ N(**0**, $\sigma_{p_{I}}^{2}$), where $\sigma_{p_{I}}^{2}$ is the indirect sire–dam variance, $Z_{I_{p}}$ is an incidence matrix with “1” in the column for the sire and in the column for the dam of the group mate of the focal individual, and e is the vector of random residuals, with e ~ N(**0**, $\sigma_{e}^{2}$).

## 3 RESULTS

### 3.1 Variability models

Both direct and indirect estimated sire effects for variability showed near‐zero correlations with simulated breeding values of sire for growth ($A_{\mathrm{GR}}$, Tables 4 and 5). Therefore, variability models do not capture genetic variation in the trait level, which is expected.

**TABLE 4:** Correlations between estimated direct sire effects for variability and simulated breeding values for growth, and direct and indirect breeding values for *b*

Scenario^a^	$\bar{b}$ effect	$A_{\mathrm{GR}}$	$A_{D_{b}}$	$A_{I_{b}}$
1	Competition	0.02	−0.96	−0.15
2	Neutral	0.02	−0.96	0.04
3	Cooperation	0.02	−0.91	0.07
4	Competition	0.02	−0.98	−0.05
5	Neutral	0.02	−0.98	−0.02
6	Cooperation	0.02	−0.96	0.02
7	Competition	−0.01	−0.80	−0.33
8	Neutral	0	−0.80	−0.04
9	Cooperation	0	−0.60	0.19
10	Competition	0	−0.80	−0.46
11	Neutral	0	−0.87	−0.22
12	Cooperation	0	−0.85	0.05
13	Competition	−0.01	−0.97	−0.03
14	Neutral	−0.01	−0.96	0.01
15	Cooperation	−0.01	−0.91	0.05
16	Competition	0	−0.96	−0.14
17	Neutral	0	−0.96	−0.02
18	Cooperation	0	−0.91	0.09
19	Competition	0.01	−0.96	−0.16
20	Neutral	0.01	−0.96	−0.04
21	Cooperation	0.01	−0.91	0.07

^a^ Details of the scenarios are summarized in Table 2.

**TABLE 5:** Correlations between estimated indirect sire effects for variability and simulated breeding values for growth, and direct and indirect breeding values for *b*

Scenario^a^	$\bar{b}$ effect	$A_{\mathrm{GR}}$	$A_{D_{b}}$	$A_{I_{b}}$
1	Competition	0.01	−0.15	−0.93
2	Neutral	0.01	−0.04	−0.91
3	Cooperation	0.01	0.08	−0.84
4	Competition	0.02	−0.43	−0.81
5	Neutral	0.02	−0.17	−0.87
6	Cooperation	0.01	0.15	−0.83
7	Competition	−0.01	−0.04	−0.94
8	Neutral	−0.01	0	−0.90
9	Cooperation	−0.01	0.03	−0.84
10	Competition	−0.02	−0.04	−0.98
11	Neutral	−0.02	−0.01	−0.98
12	Cooperation	−0.02	0.03	−0.97
13	Competition	0	−0.26	−0.69
14	Neutral	0	0	−0.61
15	Cooperation	0	0.19	−0.47
16	Competition	0	−0.12	−0.93
17	Neutral	0	−0.01	−0.92
18	Cooperation	0	0.11	−0.85
19	Competition	0	−0.15	−0.93
20	Neutral	0	−0.04	−0.91
21	Cooperation	0	0.07	−0.85

^a^ Details of the scenarios are summarized in Table 2.

#### 3.1.1 Direct effects

The estimated direct sire effects on variability showed strong negative correlations with simulated direct breeding values for *b* $(A_{D_{b}},$ resistance to competition) under competition, cooperation, and for neutral *b* (Table 4). Therefore, offspring of sires that are resistant to competition (i.e., have higher *b*) show lower variability of body weight. Correlations between estimated direct sire effects and simulated indirect breeding values for *b* ($A_{I_{b}}$), on the other hand, were near zero, under competition, cooperation, and for neutral *b*. These results indicate that cooperative effects of sires ($A_{I_{b}}$) have negligible effect on the phenotypic variation among their offspring. In conclusion, these results suggest that current (i.e., direct) models of inherited variability capture mostly the direct genetic effects ($A_{D_{b}}$) of competition, but not the indirect effect ($A_{I_{b}}$). In other words, they capture the sensitivity of individuals to competition, but not the competitive effects of individuals on the phenotypes of their group mates.

With a higher direct genetic variance in *b* ($\sigma_{A_{D}};$ compared to the default value), or lower indirect genetic variance in *b* ($\sigma_{A_{I}}$), correlations between estimated direct sire effects and simulated direct breeding values for *b* ($A_{D_{b}}$) were slightly closer to −1. The opposite was true for lower direct genetic variance in *b* ($\sigma_{A_{D}}$) and higher indirect genetic variance in *b* ($\sigma_{A_{I}}$). When the direct genetic variance in *b* was small, or when the indirect genetic variance in *b* was large, the direct model for inherited variability captured more indirect genetic effects, resulting in higher negative correlations between estimated direct sire effects and simulated indirect breeding values for *b* ($A_{I_{b}}$), Table 4).

#### 3.1.2 Indirect effects

Correlations between estimated indirect sire effects on variability and simulated indirect breeding values for *b* ($A_{I_{b}}$) were strongly negative, in competition, cooperation, and neutral scenarios (Table 5). This result indicates that group mates of offspring of sires that have high $A_{I_{b}}$, that is, sires that are cooperative, have lower variability. Similar to the previous model, correlations between estimated indirect sire effects and simulated direct breeding values for *b* ($A_{D_{b}}$) were small and negative under competition, and close to zero under cooperation and for neutral *b*. Thus, indirect models of inherited variability capture mostly indirect genetic effects of competition, but not the direct effects ($A_{D_{b}}$). In other words, they capture the competitive effects of individuals on the phenotypes of their group mates, but not the sensitivity of individuals to competition.

With a higher indirect genetic variance in *b* ($\sigma_{A_{I}},$), the correlation between estimated indirect genetic effects of a sire and indirect breeding values for *b* was closer to −1. When $\sigma_{A_{I}}$ was low or when direct genetic variance in *b* ($\sigma_{A_{D}}$) was high, correlations between estimated indirect genetic effects of a sire and simulated direct breeding values for *b* slightly increased.

### 3.2 Trait models

Correlations between estimated sire and dam effects for growth, from both direct and indirect sire–dam models for trait values and simulated direct and indirect breeding values for *b* were near 0 (results not shown). Trait models, therefore, do not capture genetic effects of competition generated by the model in Equations 1 and 2. This result is not surprising, as the classical sire–dam model does not include IGEs, while the indirect sire–dam model is essentially the variance‐component version of an IGE model, which does not make a connection between the level of IGEs and trait variability.

#### 3.2.1 Direct effects

Estimated direct sire and dam effects for growth showed a strong positive correlation with simulated direct breeding values (~0.83) for all scenarios (results not shown). Correlations were lower than 1 because dam effects were based on only 10 observations; Correlations were near 1 when considering sires only (results not shown).

#### 3.2.2 Indirect effects

Estimated indirect sire and dam effects showed a moderately negative correlation (−0.33) with simulated breeding values for growth under competition, but a moderate and positive correlation (0.26) under cooperation. Hence, the sign of the correlation corresponded to the sign of $\bar{b}$. Thus, individuals with high genetic potential for growth reduce the growth of their group mates under competition but increase the growth of their group mates under cooperation. Changes in values of $\sigma_{A_{\mathrm{GR}}}$, $\sigma_{A_{I}}$, and $\sigma_{A_{D}}$ had only a minor effect on the estimated correlations.

### 3.3 Genetic correlations between breeding values

The above reported results are based on data where genetic correlations between simulated breeding values were 0. We also investigated scenarios with correlations of −0.5 or +0.5 between breeding values (with default values for the other parameters). Results are in Tables S1–S4. As expected, estimated correlations between genetic effects from the direct sire model and $A_{I_{b}}$ and $A_{\mathrm{GR}}$ increased, when $A_{D_{b}}$ had a non‐zero correlation with $A_{I_{b}}$ and $A_{\mathrm{GR}}$ (Table S1). Similarly, an increase in estimated correlations was observed between genetic effects from the indirect sire model, when $A_{I_{b}}$ had a non‐zero correlation with $A_{D_{b}}$ and $A_{\mathrm{GR}}$ (Table S2), and in trait models when $A_{\mathrm{GR}}$ had a non‐zero correlation with $A_{D_{b}}$ and $A_{I_{b}}$.

## 4 DISCUSSION

We investigated whether current statistical models for inherited variability and for trait values capture direct and indirect genetic effects of competition on variability. Our results show that the ordinary direct model of inherited variability almost entirely captures the direct genetic effect of competition on variability, as illustrated by large correlations between estimated genetic effects and simulated direct breeding values for *b*. Similarly, an indirect model of inherited variability captures the indirect genetic effects of competition. Models for trait levels, however, capture only a small part of the genetic effects of competition.

### 4.1 Capturing *b*

**FIGURE 1:**
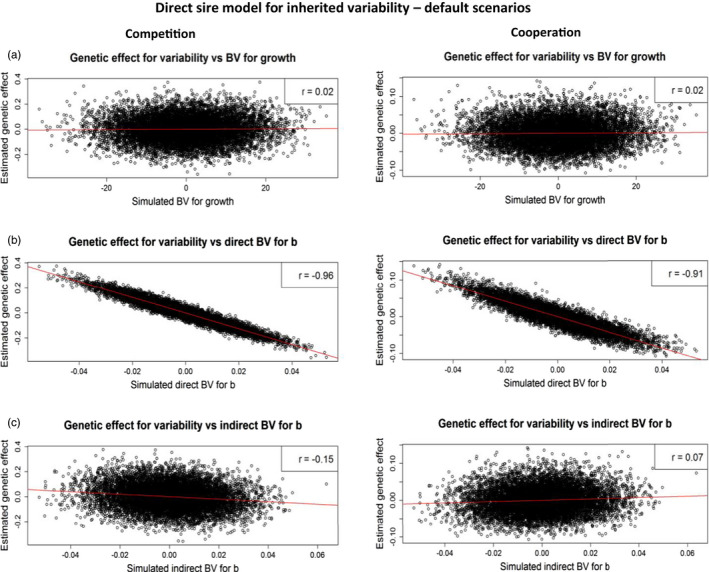
Correlations between estimated direct genetic effects of a sire for variability and simulated direct breeding values of a sire for growth (a), simulated direct breeding values of a sire for *b* (b), and simulated indirect breeding values of a sire for *b* (c) under competition and cooperation

In Marjanovic et al. (2018), we developed a quantitative genetic model (Equations 1 and 2) in which the regression coefficient *b* comprises both a direct and an indirect genetic effect. Using simulations, we demonstrated that IGEs and variability can co‐evolve, because the regression coefficient can respond to selection, similar to in trait‐based IGE models (Chenoweth et al., 2010). Therefore, both direct and indirect genetic effects on *b* affect phenotypic variability. In the current direct quantitative genetic models for inherited variability, the contribution of the social partner is ignored, which is illustrated by the results of this study, where the direct sire model for inherited variability failed to capture indirect genetic effects on *b*. In contrast, the relationship between estimated genetic effects of a sire for variability and simulated direct genetic effects for *b* showed a consistently linear relationship (Figure 1). Response to selection for higher uniformity, relying on direct genetic effect only, may be less effective as an entire level of potential genetic variation (the cooperative effect $A_{I_{b}}$) is not exploited. In addition, the presence of IGEs on *b* may cause a response in variability to divergence from its expectation, particularly when they are correlated to direct genetic effects on *b* (Ellen et al., 2014).

**FIGURE 2:**
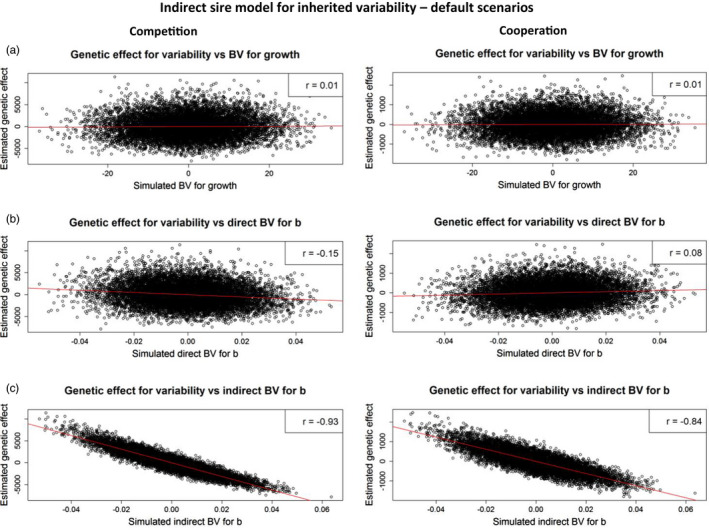
Correlations between estimated indirect genetic effects of a sire for variability and simulated direct breeding values of a sire for growth (a), simulated direct breeding values of a sire for *b* (b), and indirect breeding values of a sire for *b* (c), under competition and cooperation

When traits are affected by social interactions, selection strategies that account for both direct and indirect genetic effects can result in a higher response to selection (e.g., Bijma et al., 2007; Griffing, 1976; Muir, 1996). Future breeding programs aiming to reduce variability may, therefore, need to consider both direct and indirect genetic effects. Using an indirect sire model for inherited variability, we showed that estimated genetic effects of a sire had a high correlation with the simulated indirect breeding values for *b*. Also, this relationship is remarkably linear (Figure 2).

Capturing genetic effects of competition on variability, therefore, is promising with models for inherited variability, but may require a two‐step analysis, in which direct and indirect genetic effects are estimated separately, and subsequently combined into a total breeding value for variability, analogous to IGE models for trait values (Bijma et al., 2007). The benefit of such an approach is that it only requires group‐structured data, but not time‐series data, as the analysis is performed on the final phenotype.

The use of a one‐step approach to estimate direct and indirect breeding values for *b* would be challenging with the experimental design used in this study, where groups consisted of two individuals and offspring of a sire were randomly assigned to groups. Since each individual was both a focal individual and a social partner, calculation of the direct and social within‐family variance would require using all data twice. In other words, the same data would be needed to calculate the variance among the offspring of each sire and to calculate the variance among the social partners of the offspring of each sire. In the present study, we followed the experimental design of Marjanovic et al. (2018), which has groups of only two individuals. However, the need for a two‐step analysis can be avoided by using larger groups consisting of members of two families each. In such a design, the y‐variable could be the within‐group variance of each family in the group (two records per group), and both a direct genetic effect of the family and an indirect genetic effect of the partner family could be fitted. Alternatively, if multiple observations of body weight of two individuals in a group are available, direct and indirect genetic components of *b* could be estimated using a random regression method (Marjanovic et al., 2018).

### 4.2 Validation experiments

To validate the results of this study and the previous study by Marjanovic et al. (2018), the proposed models should be tested on empirical data. Empirical data could give insight into whether the theoretical possibility that IGEs contribute to genetic variation in variability are also biologically relevant, and in which situations. In addition, it would allow testing the statistical models proposed here and to optimize methods and models for future studies aiming to estimate genetic effects of competition. Selection experiments where one selection strategy involves selection for direct genetic effects on variability only, while the other would select for both direct and indirect genetic effects, would also allow quantifying the contribution of IGEs to response to selection in variability.

The experiments could have a group structure with two individuals per group, similar to our study, but trials involving larger group sizes could also be conducted to test the single‐step analysis suggested above and to quantify the effect of group size on the estimates. For groups of two individuals, data on both individuals in each group should ideally be collected at several time points. Such time‐series data would allow using not only a random regression approach as suggested by Marjanovic et al. (2018), but also the direct model and the indirect model for inherited variability presented in this study could be used. A combination of time‐series analysis and analysis of the final record would give insight into the mechanisms underlying inherited variability. While the presence of inherited variability has been demonstrated convincingly in several cases (Hill & Mulder, 2010), little is known of the underlying mechanisms, and the social environment has received little attention in such studies. Such experiments could be performed using zebrafish as a model organism, as this species shows substantial competition and fast growth.

For estimation of direct and indirect breeding values for *b* in a commercial setting in plant and animal breeding, new phenotyping techniques that involve automated phenotype detection and video tracking of individuals in 3D space could be used in the future (see, e.g., idTracker, http://www.idtracker.es/). These techniques can provide large‐scale time‐series data on individual trait values (e.g., body weight calculated from the 3D image, size, and shape plants) and information on social interactions between individuals. The combination of such technologies with new models for interactions among individuals, such as Equations 1 and 2, facilitates the integration of the social genetic environment into quantitative genetic descriptions of inheritance and response to selection.

## 5 CONCLUSION

Our results show that a direct model of inherited variability almost entirely captures the genetic sensitivity of individuals to competition, while an indirect model of inherited variability captures the cooperative genetic effects of individuals on their partners. Models for trait levels, however, capture only a small part of the genetic effects of competition. The estimation of direct and indirect genetic effects of competition, therefore, is possible with models for inherited variability but may require a two‐step analysis or a different data setup involving larger groups.

## CONFLICT OF INTEREST

The authors declare that they have no competing interests.

## Supporting information

Table S1–S4

## Data Availability

This article uses simulation data for which input parameters and detailed steps are presented in Section 2 section. Simulation data and scripts are available from the authors on request.
