# Supplementary material for: Capturing indirect genetic effects on phenotypic variability: Competition meets canalization
Source: Evol Appl. 2022 Mar 8;15(4):694–705. doi: 10.1111/eva.13353 (PMC9046766; doi:10.1111/eva.13353)
Supplement: Supplementary file 1 — Table S1–S4 [file EVA-15-694-s001.pdf]

Supplementary material

Table S1. Estimated correlations between simulated breeding values and estimated genetic effects, when simulated breeding values are genetically correlated

Direct sire model for inherited variability

Correlation 0.5 between simulated A_{D_b} and A_{I_b}

		A_{GR}	A_{D_b}	A_{I_b}
$\hat{A}_{DV,s}$	competition	0.0	-0.96	-0.61
	cooperation	0.0	-0.90	-0.31

Correlation -0.5 between simulated A_{D_b} and A_{I_b}

		A_{GR}	A_{D_b}	A_{I_b}
$\hat{A}_{DV,s}$	competition	0.0	-0.95	0.32
	cooperation	0.0	-0.91	0.53

Correlation 0.5 between simulated A_{GR} and A_{D_b}

		A_{GR}	A_{D_b}	A_{I_b}
$\hat{A}_{DV,s}$	competition	-0.59	-0.95	-0.20
	cooperation	-0.64	-0.90	0.06

Correlation -0.5 between simulated A_{GR} and A_{D_b}

		A_{GR}	A_{D_b}	A_{I_b}
$\hat{A}_{DV,s}$	competition	0.58	-0.95	-0.18
	cooperation	0.64	-0.90	0.08

Correlation 0.5 between simulated A_{GR} and A_{I_b}

		A_{GR}	A_{D_b}	A_{I_b}
$\hat{A}_{DV,s}$	competition	0.0	-0.95	-0.11
	cooperation	0.0	-0.91	0.05

Correlation -0.5 between simulated A_{GR} and A_{I_b}

		A_{GR}	A_{D_b}	A_{I_b}
$\hat{A}_{DV,s}$	competition	0.0	-0.96	-0.16
	cooperation	0.0	-0.91	0.05

Table S2. Estimated correlations between simulated breeding values and estimated genetic effects, when simulated breeding values are genetically correlated

Indirect sire model for inherited variability

Correlation 0.5 between simulated A_{D_b} and A_{I_b}

		A_{GR}	A_{D_b}	A_{I_b}
$\hat{A}_{IV,s}$	competition	0.0	-0.56	-0.93
	cooperation	0.0	-0.33	-0.81

Correlation -0.5 between simulated A_{D_b} and A_{I_b}

		A_{GR}	A_{D_b}	A_{I_b}
$\hat{A}_{IV,s}$	competition	0.0	0.38	-0.93
	cooperation	0.0	0.52	-0.86

Correlation 0.5 between simulated A_{GR} and A_{D_b}

		A_{GR}	A_{D_b}	A_{I_b}
$\hat{A}_{IV,s}$	competition	0.19	-0.01	-0.91
	cooperation	0.34	0.23	-0.78

Correlation -0.5 between simulated A_{GR} and A_{D_b}

		A_{GR}	A_{D_b}	A_{I_b}
$\hat{A}_{IV,s}$	competition	-0.20	0.0	-0.90
	cooperation	-0.36	0.24	-0.78

Correlation 0.5 between simulated A_{GR} and A_{I_b}

		A_{GR}	A_{D_b}	A_{I_b}
$\hat{A}_{IV,s}$	competition	-0.47	-0.13	-0.92
	cooperation	-0.44	0.10	-0.84

Correlation -0.5 between simulated A_{GR} and A_{I_b}

		A_{GR}	A_{D_b}	A_{I_b}
$\hat{A}_{IV,s}$	competition	0.46	-0.14	-0.93
	cooperation	0.42	0.09	-0.85

Table S3. Estimated correlations between simulated breeding values and estimated genetic effects, when simulated breeding values are genetically correlated

Direct sire-dam model for the trait

Correlation 0.5 between simulated A_{D_b} and A_{I_b}

		A_{GR}	A_{D_b}	A_{I_b}
$\hat{A}_{I_{V,s}}$	competition	0.83	0.0	0.0
	cooperation	0.84	0.0	0.0

Correlation -0.5 between simulated A_{D_b} and A_{I_b}

		A_{GR}	A_{D_b}	A_{I_b}
$\hat{A}_{I_{V,s}}$	competition	0.83	0.0	0.0
	cooperation	0.83	0.0	0.0

Correlation 0.5 between simulated A_{GR} and A_{D_b}

		A_{GR}	A_{D_b}	A_{I_b}
$\hat{A}_{I_{V,s}}$	competition	0.83	0.41	0.0
	cooperation	0.83	0.42	0.0

Correlation -0.5 between simulated A_{GR} and A_{D_b}

		A_{GR}	A_{D_b}	A_{I_b}
$\hat{A}_{I_{V,s}}$	competition	0.83	-0.41	0.0
	cooperation	0.83	-0.41	0.0

Correlation 0.5 between simulated A_{GR} and A_{I_b}

		A_{GR}	A_{D_b}	A_{I_b}
$\hat{A}_{I_{V,s}}$	competition	0.83	0.0	0.41
	cooperation	0.83	0.0	0.42

Correlation -0.5 between simulated A_{GR} and A_{I_b}

		A_{GR}	A_{D_b}	A_{I_b}
$\hat{A}_{I_{V,s}}$	competition	0.83	0.0	-0.41
	cooperation	0.83	0.0	-0.41

Table S4. Estimated correlations between simulated breeding values and estimated genetic effects, when simulated breeding values are genetically correlated

Indirect sire-dam model for the trait

Correlation 0.5 between simulated A_{D_b} and A_{I_b}

		A_{GR}	A_{D_b}	A_{I_b}
$\hat{A}_{IV,s}$	competition	-0.33	0.0	0.0
	cooperation	0.27	0.0	0.0

Correlation -0.5 between simulated A_{D_b} and A_{I_b}

		A_{GR}	A_{D_b}	A_{I_b}
$\hat{A}_{IV,s}$	competition	-0.33	0.0	0.0
	cooperation	0.28	0.0	0.0

Correlation 0.5 between simulated A_{GR} and A_{D_b}

		A_{GR}	A_{D_b}	A_{I_b}
$\hat{A}_{IV,s}$	competition	-0.33	-0.16	0.0
	cooperation	0.28	0.13	0.0

Correlation -0.5 between simulated A_{GR} and A_{D_b}

		A_{GR}	A_{D_b}	A_{I_b}
$\hat{A}_{IV,s}$	competition	-0.33	0.15	0.0
	cooperation	0.28	-0.14	0.0

Correlation 0.5 between simulated A_{GR} and A_{I_b}

		A_{GR}	A_{D_b}	A_{I_b}
$\hat{A}_{IV,s}$	competition	-0.33	0.0	-0.17
	cooperation	0.28	0.0	0.13

Correlation -0.5 between simulated A_{GR} and A_{I_b}

		A_{GR}	A_{D_b}	A_{I_b}
$\hat{A}_{IV,s}$	competition	-0.33	0.0	0.16
	cooperation	0.28	0.0	-0.14
